# Treatment of Infiltrative Superficial Tumors in Awake Standing Horses Using Novel High-Frequency Pulsed Electrical Fields

**DOI:** 10.3389/fvets.2019.00265

**Published:** 2019-08-14

**Authors:** Christopher R. Byron, Matthew R. DeWitt, Eduardo L. Latouche, Rafael V. Davalos, John L. Robertson

**Affiliations:** ^1^Department of Large Animal Clinical Sciences, Virginia-Maryland Regional College of Veterinary Medicine, Virginia Tech, Blacksburg, VA, United States; ^2^Virginia Tech-Wake Forest School of Biomedical Engineering and Science, College of Engineering, Virginia Tech, Blacksburg, VA, United States

**Keywords:** horse, cutaneous tumors, electroporation, H-FIRE, models of human disease, focal ablation, non-thermal tumor ablation

## Abstract

Irreversible electroporation is a proven ablation modality for local ablation of soft tissue tumors in animals and humans. However, the strong muscle contractions associated with the electrical impulses (duration, 50–100 μs) requires the use of general anesthesia and, in most situations, application of neuromuscular blockade. As such, this technology is not used in an outpatient setting for ablating common cutaneous tumors (e.g., squamous cell carcinoma or melanoma) in humans or animals. Recently, high-frequency irreversible electroporation (H-FIRE) technology has been developed to enable electroporation of tumors without stimulation of nearby skeletal muscle. H-FIRE administers bursts of electrical pulses (duration, 0.5–2 μs) through bipolar electrodes placed in tumor parenchyma. We hypothesized that H-FIRE could be used to safely ablate superficial tumors in standing, awake horses without the need for general anesthesia. Here, we describe the treatment of superficial tumors in five horses using this novel ablation therapy without the need for general anesthesia. In each case, H-FIRE therapy predictably ablated tumor volume. All patients tolerated the procedure, no complications developed, and veterinary personnel safety was maintained. The H-FIRE treatment may be useful for treatment in veterinary and human patients in an outpatient setting without the need for hospitalization, general anesthesia, and advanced monitoring techniques.

## Introduction

Cutaneous tumors, including sarcoid, squamous cell carcinoma, and melanoma, are extremely common in horses (1, 2). While many small, circumscribed lesions can be controlled with surgical resection, cryoablation, hyperthermy, or localized chemotherapy (e.g., topical 5-fluorouracil or cisplatin in oil or bioabsorbable beads), infiltrative lesions are typically difficult to control. Complete resolution may not be obtained because local infiltration around critical structures (nerves, major blood vessels) precludes surgical removal, or because of extensive local tissue invasion by tumors and/or the presence of recurrent, locally-aggressive growth of tumors and multiple tumor sites. The use of multiple treatment modalities, such as a combination of surgical resection and local chemotherapy, is common in attempting therapy of such tumors. Horses with advanced disease frequently are not treated and may be euthanized when quality of life or function decreases. In addition, many treatments must be performed during multiple general anesthetic procedures. Taken together, all of these limitations indicate a pressing need for development of novel methods for management of such tumors.

In an effort to improve treatment outcomes, electrochemotherapy has been used recently for treatment of tumors in veterinary patients (3). This therapeutic approach combines direct delivery of electric pulses with local delivery of chemotherapeutics; the application of electrical current temporarily permeabilizes membranes and increases delivery of drugs to tumor cells. This use of electroporation enhances cytotoxicity of chemotherapeutic drugs up to 5-fold (4), potentially allowing use of lower and less-toxic dose protocols. Electrochemotherapy has been used in the treatment of various cutaneous tumors of horses (5). This treatment has most commonly been applied to equine sarcoid tumors (6, 7) with long-term recurrence rates <10% reported. Reports of treatment for other tumors, including melanoma (8), are scant and wide applicability of results is limited by the small number of reports and few included animals. Disadvantages of electrochemotherapy include the need for cytotoxic chemotherapeutic drugs, necessity of multiple general anesthesia episodes, and treatment-associated induction of vigorous skeletal muscle contraction that can pose a potential hazard to patients and medical personnel. In some settings, treatments may require intensive monitoring, including administration of neuromuscular paralytic drugs and synchronization of electrical treatment impulses with the cardiac cycle (9).

Irreversible electroporation (IRE), or the application of high voltage, short duration (50–100 μs) electric fields for non-thermal tumor ablation, is a potentially useful tool for treatment of cancer (10). This technology differs from electrochemotherapy in that with IRE, membranes are irreversibly damaged by electrical pulses, leading to cell death without the need for chemotherapeutic drugs. IRE treatments produce negligible local tissue heating (<5°C) allowing for treatment near critical structures such as nerves and vasculature. In this regard, IRE is distinct from other energy-intensive ablation modalities (radiofrequency or microwave) that ablate tissues by generating tumor heating (typically >50°C). IRE has been used clinically for treatment of various cancers (hepatic, pancreatic, and prostatic carcinomas) in human patients (11, 12). However, widespread clinical use of IRE has been limited by the relatively small tumor volumes that can be treated and the requirement for general anesthesia and chemical neuromuscular blockade to prevent intense muscle contractions associated with the application of monopolar electric pulses. High-frequency irreversible electroporation (H-FIRE) is an advanced and novel type of IRE for non-thermal tumor ablation (13). H-FIRE uses bursts of 1–10 μs electric pulses, applied through minimally-invasive bipolar electrodes (diameter, 1–1.5 mm) inserted into the targeted region, to generate electric fields in excess of 1,000 V/cm, an energy field which destroys tumor cells. The nature of the treatment is such that H-FIRE does not cause generalized muscle contraction and neither general anesthesia nor neuromuscular blockade/paralytics are needed. H-FIRE therapy has been used for treatment of intracranial meningioma in dogs (14). The combination of selective tumor cell ablation, ease of treatment (minimally-invasive application and no requirement for general anesthesia/neuroparalytics), and sparing of critical anatomic structures are all potentially advantageous for treatment of cutaneous tumors in horses.

We hypothesized that H-FIRE could be safely used for the treatment of cutaneous tumors in standing, awake sedated horses. Our study investigated the safety and clinical benefit of H-FIRE (electric pulses of 2 μs duration and up to 3,100 V) for treatment of horses with common cutaneous neoplasms, without the need for general anesthesia, neuromuscular blockades, or cardiac synchronization of impulse delivery.

## Materials and Methods

### Animals

Five horses with naturally-occurring multifocal cutaneous tumors were prospectively included in the study. This was designed as a Phase I clinical trial to evaluate the safety and clinical efficacy of H-FIRE in the treatment of cutaneous tumors in horses in an outpatient setting. Potential patients were identified from the caseload of the large animal hospital and also recruited via an announcement on the college clinical trials website. All animals were treated at the Harry T. Peters Large Animal Hospital at Virginia Tech, Blacksburg, VA.

All patients were diagnosed to have surgically non-resectable cutaneous tumors (Table 1). Prior to treatment, punch biopsies (diameter, 3 mm) were obtained (using local anesthesia/nerve block) and tumors were histologically characterized by a board-certified veterinary pathologist. Horses were deemed systemically healthy via physical examination, serum biochemistry, and hemogram test results. For inclusion in the study, horses had no other clinically significant localized disease. All horses had at least one tumor accessible for insertion of treatment electrodes which could be serially photographed and assessed with ultrasound imaging and measured to assess treatment effects. Horses with melanoma had tumors in multiple areas; for these horses, solitary melanomas in a single accessible site were treated. All study protocols were approved by the Virginia Tech Institutional Animal Care and Use Committee (protocol # 13-144-CVM) and informed consent for this experimental therapy was obtained from owners prior to enrollment/treatment.

**Table 1:** Data regarding 5 horses that underwent treatment for superficial tumors with high-frequency irreversible electroporation.

**Patient**	**Age at treatment (years)**	**Tumor type**	**No. of treatments**	**Initial tumor size (cm)**	**Final tumor size (cm)**	**% Change in tumor volume**
1	11	Multifocal melanoma	4	5.0 × 2.5 × 2.5	4.0 × 2.0 × 2.0	−52.1
2	14	Perivulvar squamous cell carcinoma	2	3.5 × 3.5 × 3.5	3.0 × 3.0 × 1.6	−66.4
3	20	Perianal squamous cell carcinoma	2	6.0 × 8.0 × 4.0	1.0 × 0.5 × 0.5	−99.8
4	19	Intranasal mast cell tumor	2	3.0 × 1.5 × 1.0	2.2 × 1.5 × 1.0	−26.7
5	12	Mulifocal melanoma	3	5.1 × 4.5 × 2.5	1.5 × 0.5 × 0.25	−99.2

### H-FIRE Parameter Selection

**Figure 1:**
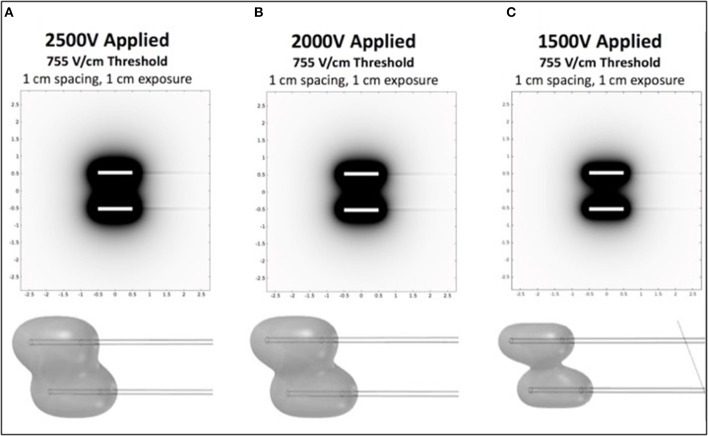
Computational COMSOL model for prediction of treatment volume in H-FIRE treatment of surface tumors. Data is shown in 2D for 1cm spacing and 1cm exposure of electrodes for 2,500 V **(A)**, 2,000 V **(B)**, and 1,500 V **(C)**. 3D representations of the volumes are shown below each panel.

To investigate appropriate treatment voltages and the parameters required to create a contiguous ablation site using this H-FIRE system, finite element models (COMSOL Version 5.2a, COMSOL Inc., Palo Alto, CA, USA) were used to simulate treatments with two needle electrodes spaced 1 cm apart (Figure 1). Previous *in vitro* experiments predicted cell death at an electric field threshold of 500 V/cm for traditional IRE pulses and 755 V/cm for equivalent energy H-FIRE treatments with 2 μs pulses. A 2 cm diameter spherical simulation domain with electrical conductivity of 0.063 S/m (Siemens/meter) was used to simulate the tissue volume under treatment. The treatment electrodes were represented by two 1 mm diameter 1 cm long cylinders with electrical conductivity 4.032 × 10^6^ S/m that were placed at the center of the tissue domain and separated by 1 cm edge-to-edge. The insulated electrode shafts were represented by two additional 1 mm diameter 5 cm long cylinders with electrical conductivity of 1 × 10^−12^ S/m. The electrodes and insulated shafts were meshed using the default “extra fine” preset. The tissue domain was meshed using the “normal” preset with the maximum growth rate changed from the default 1.5–1.1. The final mesh contained 1,981,031 domain elements and required ~2 min to solve on a dual core Intel i7 processor with 16 GB RAM for each voltage investigated. Treatment planning was not accomplished on a patient-by-patient basis but rather was utilized to select approximate electrical settings (1,700–3,000 V) applied to the two electrodes in order to adequately treat the various lesion sizes.

Prior to each H-FIRE treatment, modeling software results indicated the energy required to ablate predicted treatment volumes for tumors at 2,500, 2,000, and 1,500 V of energy (Figure 1). This computational modeling indicated that high voltages (>2,000 V) are required to achieve effective treatment areas (2 cm^2^) with entire treatment volumes between electrodes; this was depicted as 3D representations of the treatments. Computational models were then utilized for planning probe spacing, voltage, and electrode exposure to ensure optimal tumor ablation based on the morphology of the lesions using a look-up table of predicted treatment volumes. Custom treatment plans were implemented based on anatomical limitations for probe spacing and depth and were developed to ensure only non-thermal ablation of tumor tissue, sparing normal non-tumor tissue in adjacent anatomic areas.

### H-FIRE Treatment Protocol

All patients were treated with H-FIRE energy every 2 weeks, during 2–4 outpatient visits. Prior to treatment, tumor boundaries were visualized via ultrasonography and volumes estimated (length x width x depth) from images. For treatment, horses were sedated with detomidine (10 μg/kg, IV) and butorphanol (0.1 mg/kg, IV). Treatment sites were prepared with povidone-iodine scrub. Tumors and surrounding tissues were desensitized by local injection of lidocaine or mepivacaine (2%) into the surrounding tissues. Standard electrocardiogram leads were placed for monitoring of heart rate and rhythm. Previous studies of H-FIRE (14) showed that cardiac synchronization of electric impulses was not required, given the H-FIRE waveform configuration/duration and distance to the patient's heart. Patient attitude and responsiveness was monitored by experienced large animal technicians/nurses during procedures.

**Figure 2:**
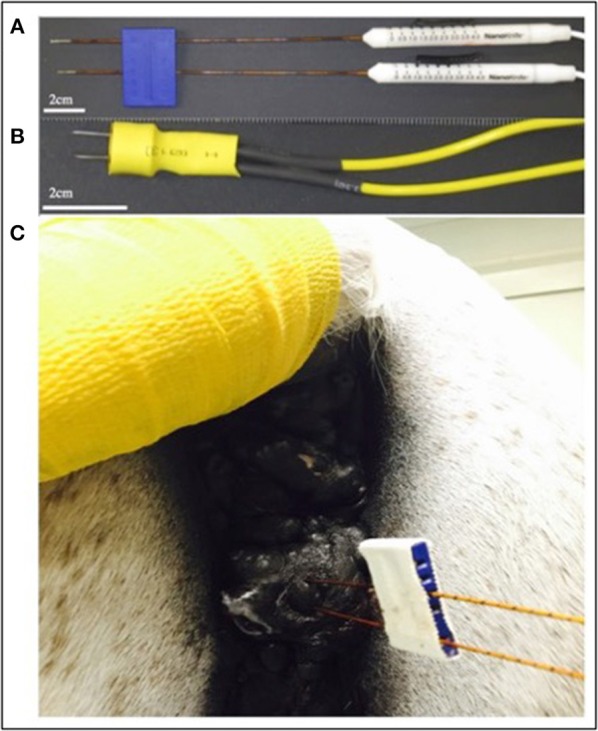
Photographs of commercially available electrodes used for H-FIRE **(A)**, custom needle electrodes used to treat small lesions **(B)**, and electrode placement in a perineal melanoma undergoing H-FIRE treatment **(C)**.

Commercially available probes (Nanoknife, Angiodynamics, Latham, NY, USA) or custom stainless steel electrodes (McMaster Carr, Douglasvill, GA, USA) were used to deliver bipolar pulses from a custom built H-FIRE pulse generator (Virginia Tech, Blacksburg, VA, USA) during all treatments. When clinical Nanoknife electrodes were utilized to treat larger lesions (>2 cm), the two electrodes were placed parallel to the cutaneous tumor with 1–1.5 cm spacing using the commercial probe spacer (Figure 2). When lesions were <2 cm, the custom electrode pair (Figure 2) was utilize to avoid arching from skin folds in the smaller lesions. The custom electrodes had an electrode spacing of 0.5 cm and treatment parameters were selected utilizing computational modeling as described previously. Exact probe placement varied based on primary tumor location, geometry, and proximity to associated critical structures and treatment parameters were selected to ensure entire lesions were exposed to electric fields greater than approximately 750 V/cm (Figure 1).

**Figure 3:**
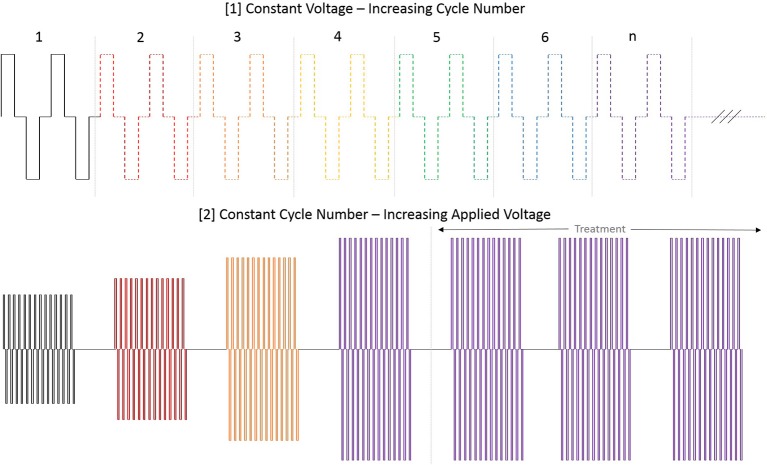
Diagram depiction of H-FIRE pre-treatment energy escalation scheme. To ensure safety and tolerability of the applied pulses, the energy is first applied at low (500 V) potential and the number of cycles is increased until desired burst duration. Subsequently, the voltage is raised until final applied potential is reached and therapy is then delivered at 1 burst per second (1 Hz).

At initiation of treatment for each tumor location, an opening pre-treatment pulse protocol was applied in order to assess patient comfort, the potential for muscle stimulation, and overall tolerance for treatment at lower applied potentials. In this protocol, the voltage was initially set to 500 V and the H-FIRE system was set to deliver a 2-5-2 waveform which consisted of a 2 μs positive pulse, a 5 μs delay, followed by a 2 μs negative pulse. If no muscle stimulation was seen and the energy was tolerated by the standing patient then the pulse train was lengthened until each burst consisted of 100 μs energized time at 500 V. The voltage was then increased in 500 V steps until the therapeutic settings were reached. An overall depiction of the pre-treatment energy escalation scheme used to determine patient safety and toleration can be seen in Figure 3.

**Figure 4:**
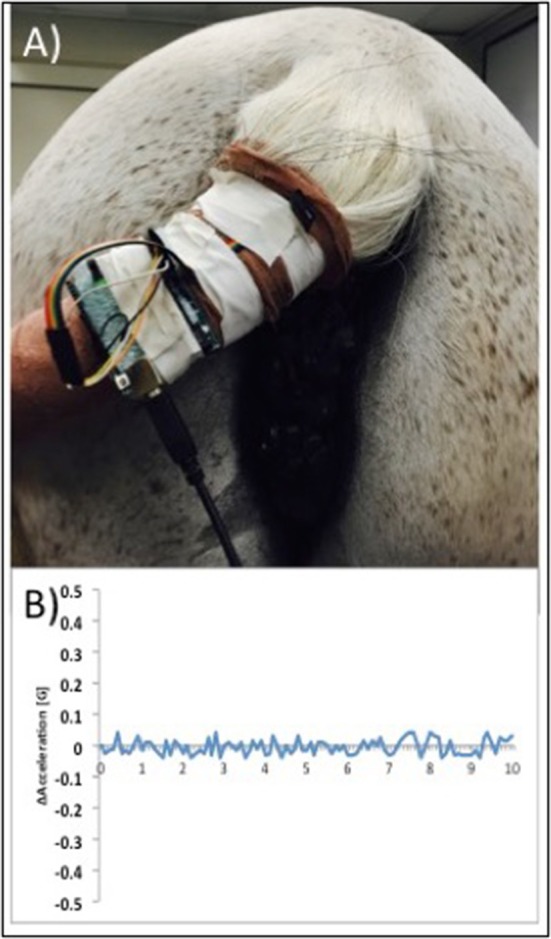
Photograph illustrating placement of a custom accelerometer to measure muscle contractions in real time during H-FIRE treatment **(A)** and example data showing minimal muscle contractions during H-FIRE treatment with 3,100 V delivered at 1Hz **(B)**.

Following dose escalation at each site, the H-FIRE system was set to deliver the 2-5-2 waveform in 100 μs bursts in the target voltage range from 1,500 to 3,100 V. Each burst was delivered at 1 Hz and it should be noted that 3,100 V was the maximum output possible with the pulse generator used in this study and higher voltages may have been optimal in some patients based on the size of lesion presented. Any treatment-associated muscle contraction was detected using a custom accelerometer, secured loosely to the skin, and confirmed with videography. Accelerometer data was collected and the acceleration per time was determined and graphed (Figure 4).

After treatment, flunixin meglumine (1.1 mg/kg, IV) was administered and patients were discharged. Owners were instructed to clean the treated areas daily and note changes in tumor size, as well as any discharge from treated sites or evidence of skin breakdown. H-FIRE treatment efficacy was evaluated during recheck examinations (prior to each successive treatment) via physical examination, surface measurement of tumor size, and ultrasonography (including measurement of tumor depth). After the final treatment, biopsy samples (diameter, 3 mm) of tumors were obtained and submitted for histology (except for one horse with an intranasal mast cell tumor, for which a post-treatment sample was not collected). Treatment end-points were determined to be when tumors either did not show further appreciable reduction in size 2 weeks after a prior treatment or had volumes of ablation >90% compared with initial tumor size or volume prior to treatment. Complete resolution of all tumors in each horse was not a goal of the study. Long-term follow-up was obtained regularly with owners via telephone and electronic communication.

## Results

### Treatments

Five horses with infiltrative tumors were successfully treated without notable treatment or post-treatment complications (Table 1). Patients had minimal reaction (involuntary muscle contraction, changes in mentation) to the treatment. All treatments were performed safely in standing horses with sedation and local anesthesia; no general anesthesia was required. Surgeon and support personnel safety was rigidly maintained at all times. During treatments, no cardiac pacing or synchronization of H-FIRE electrical pulses was required. ECG artifacts (H-FIRE-associated spike) were visible during delivery of H-FIRE electrical impulses; however, no cardiac electrical abnormalities were induced by the treatment. Involuntary skeletal muscle contractions were limited to mild local muscle stimulation as indicated by slight tail head movement (only) in all cases. To illustrate this point, representative data collected with a custom accelerometer sensor placed on the tail head during perianal treatment of a perineal region melanoma is shown in Figure 4. Data indicated no muscle contractions during a 2,500 V treatment with 100 μs burst of 2 μs bipolar pulses and minimal contractions with 3,100 V (spacing between electrodes, 1.5 cm; electrode exposure to tissue, 1 cm).

**Figure 5:**
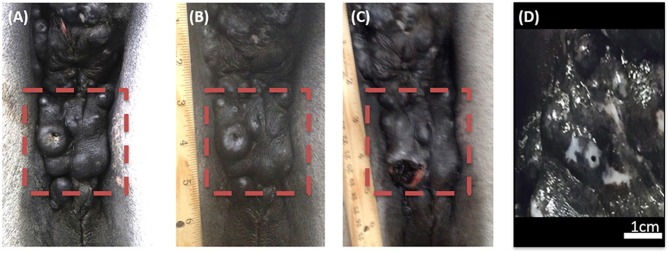
Photographic images of a perineal region melanoma before H-FIRE treatment **(A)**, immediately following a series of 2 treatments with H-FIRE **(B)**, immediately following a series of 4 treatments **(C)**, and 2 weeks following 4 treatments **(D)**. In **(D)**, notice the loss of melanin pigmentation in the treated area.

**Figure 6:**
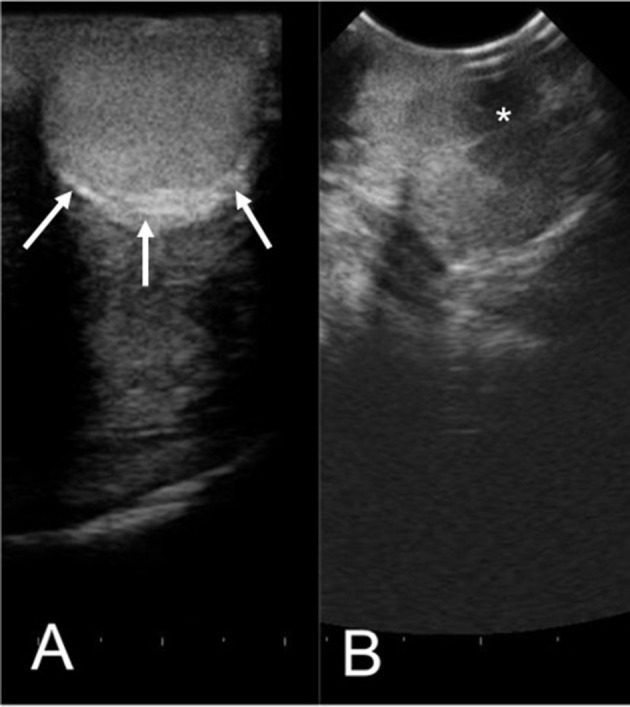
Representative ultrasonographic images of a perineal region cutaneous melanoma before **(A)** and 2 weeks after **(B)** H-FIRE treatment. In **(A)**, arrows indicate the tumor margins. In **(B)**, notice hypoechogenicity of the tumor parenchyma (asterisk). Distance between hash marks on the bottom of the figure = 5 mm.

**Figure 7:**
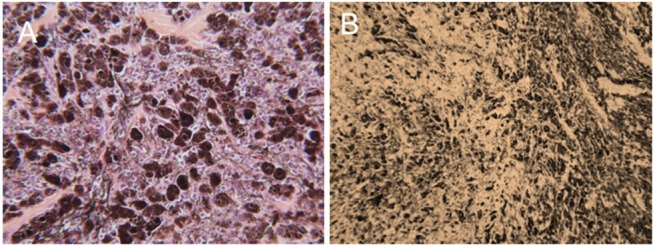
Representative photomicrographic images of perianal melanoma biopsy samples obtained from a mature, gray horse before **(A)** and after a series of 3 H-FIRE treatments **(B)**. In **(A)**, the tumor is composed of a pleomorphic population of variably-sized polygonal cells. Cells range from small round cells with sparse pigmentation to larger, more heavily pigmented cells, with brown-dark brown intracellular pigment in fine cytoplasmic granules. There are small nesting clusters of some melanocytic cells, typical of melanocytic neoplasms. Mitotic figures are uncommon, as are cells undergoing degeneration. (HE × 400). In B, treatment with H-FIRE has disrupted cells, releasing pigment, and has created zones of ablation and replacement fibrosis (HE × 200).

The efficacy of H-FIRE treatment for debulking of tumors in horses is indicated in Table 1 and Figure 5. Evaluation of ablation zone sizes determined via physical and ultrasonographic examination indicated consistent tumor volume reduction (mean, 68.8%, *SD* ± 31.39; after 2–4 treatments). A noticeable reduction of tumor volume was appreciable in each horse via gross examination. Tumor parenchyma destruction was confirmed by ultrasonographic imaging (Figure 6). In addition to reduction in tumor volume, depigmentation of skin was noticed in treatment sites of melanomas. This suggested destruction of melanin containing epithelial cells without the destruction of the underlying tissue architecture. This tissue remained depigmented after healing. However, this observation will have to be further substantiated in future studies with additional histologic evaluation. Histologic comparison of representative pre- and post-treatment tissue biopsy samples of melanomas indicated disruption of cells, release of pigment, and zones of parenchymal ablation replaced with fibrotic tissue (Figure 7). Histologic examination of post-treatment squamous cell carcinoma biopsy samples indicated areas of necrosis. Post-treatment histology was not performed for the horse with mast cell tumor.

After treatments, owners reported no systemic complications. Mild local edema was observed for up to 4 days after treatments and was managed with local wound care and anti-inflammatory agents (typically a single administration of flunixin meglumine [1.1 mg/kg, PO] or phenylbutazone [2.2 mg/kg, PO]). Long-term follow-up (2 and 3.5 years) for two horses with melanoma indicated treated tumors continued to decrease in size and did not regrow (Table 1); additionally, some tumors at distant sites continued to decrease in size. For a horse with perianal squamous cell carcinoma, follow-up examination at 5 months revealed that the tumor did not show signs of regrowth. A horse with perivulvar squamous cell carcinoma had complete resolution of the tumor 2 months after initiation of treatment. Recheck examination 3 years after treatment indicated no regrowth of the tumor. Follow-up information was not available for the horse with intranasal mast cell tumor.

## Discussion

The results of this study show that H-FIRE-mediated tumor ablation can be performed safely in standing horses with minimal risk of injury to patients or veterinary personnel. Treatments were performed in an outpatient setting without need for hospitalization, general anesthesia, neuromuscular blockade, or advanced monitoring techniques and equipment. Advantages of outpatient treatment include decreased cost and enhanced convenience for clients as well as decreased burden on hospital resources. In addition, although H-FIRE parameters have not yet been optimized, results indicated substantial reduction in treated tumor volumes. Based on our experience, this technology may have future applicability for treatment of tumors in veterinary and human patients, as several disadvantages of current IRE and electrochemotherapy protocols are avoided. The fact that H-FIRE treatments could be applied to awake, standing horses suggests this is an exciting development in tumor treatment technologies for all species. To the best of our knowledge this is the first application of pulses longer than 1 μs over 3,000 V in a clinical setting. Also, based on computer modeling, H-FIRE therapy may produce more uniform treatment areas in heterogeneous tissue (tissue with both epithelial and connective tissue components) which may have complex tissue impedance features. A uniform ablation zone should enable more accurate computational models and improved treatment planning (15).

Although many of the common histotypes of cutaneous neoplasms seen in humans have been reported in horses (squamous cell carcinoma, basal cell carcinoma, mast cell sarcoma) (1, 2), equine melanomas and sarcoids do not have close homologies in humans. For example, while it is fairly common in humans to have relatively small, solitary, infiltrative cutaneous melanomas, with a propensity to metastasize as they progress, equine melanomas show slow (in the order of years), expansile, infiltrative growth, as single or multiple nodules and do not appear prone to metastasis with increasing size. In fact, many conflicting reports in the veterinary literature, spanning well over 100 years, have debated whether equine melanoma is a benign or malignant neoplasm, or a neoplasm, at all. There is evidence that multiple types of melanocytic tumors with various clinical behaviors develop in both gray and non-gray horses (16). There is little doubt that equine melanoma is an infiltrative neoplasm, difficult to control, and that it is frequently recurrent and/or progressive following any surgical/medical therapy. In this regard, clinically, equine melanoma shares troublesome characteristics with human Stages 3–4 melanoma where complete surgical ablation may not be possible and surgical “cure” unlikely.

Equine melanoma is very common in dilute coat-color (gray) horses, and is one of the most common types of skin tumors in horses (17). In contrast, the majority of human skin tumors are not melanin-containing, and many are related to ultraviolet radiation exposure (not apparently a factor in the development of equine skin tumors); genetics and heredity in humans seems to play a minimal role in the pathogenesis of common skin tumors. However, the common occurrence of melanoma in horses makes the horse an attractive translational model for development of novel therapies like H-FIRE that may eventually be used to treat skin neoplasms in humans and other species (dogs and cats for example).

Equine sarcoids appear to be a unique disease of horses, although with histologic similarities to human fibropapilloma and fibrohistiocytic tumors of other species. Equine sarcoids are frequently refractory to standard surgical ablation but more amenable to treatment with other modalities (cryotherapy, chemotherapy, combination therapy) (18, 19). We feel that H-FIRE may be useful in treatment of recurrent, treatment-resistant cutaneous neoplasms in all species (human and otherwise) and especially useful in anatomically-complex areas where gradual tumor ablation may preserve cosmesis. The H-FIRE treatment method has potential advantages for treatment of equine sarcoids since it can be used in sedated horses without the requirement of general anesthesia, which is necessary for some other treatment methods such as electrochemotherapy. Although we did not treat equine sarcoids in this study, investigation of H-FIRE protocols for treatment of this tumor type is a future goal of the authors. The ability to treat equine sarcoids in standing horses would be an advantage in comparison with currently available electrochemotherapy treatments.

Although mast cell tumors are extremely common in dogs, they are relatively rare in horses (20). In equids, they are typically found in skin but can affect other tissues such as conjunctiva (21). To the authors' knowledge, treatment of mast cell tumors in horses with electroporation technologies has not been reported. However, electroporation treatments may have utility for treatment of these tumors in equids, since electrochemotherapy reportedly (22) compares favorably to surgical resection for mast cell tumors in dogs.

Horses are at an increased risk of complications or death as a result of general anesthesia compared with other species (23). Therefore, treatment under sedation and local anesthesia should be considered advantageous for the treatment of superficial tumors. With this in mind, reducing muscle contractions associated with IRE treatments was important. Our approach was to change electrical impulse parameters in order to reduce muscle contraction. Results of this study indicate this is a viable approach, since muscle contractions are known to increase exponentially as pulse duration increases.

There are some important limitations to this current study, including the small sample size, and absence of long-term follow up for all animals. The electrode spacing, applied voltages, and number of pulses delivered to each site was not held constant for each patient—as the primary goal of this study was to maximize treatment effect. As such, the energy delivered was changed on a per-patient basis. In addition, flunixin meglumine was administered immediately after treatment; administration before treatment may have further reduced intra- and post-procedural inflammation. However, clinical signs of inflammation were modest and were typically observed after cessation of treatment. Well-controlled prospective studies on a large number of patients will be necessary to elucidate which treatment parameters play the largest role in treatment outcomes. Treatments did not result in complete regression of all tumors. Likewise, prior studies utilizing IRE did not obtain 100% regression rates and more powerful computational pre-treatment planning must be employed to assure complete target tissue destruction to reduce tumor regrowth (24). The promising results of the current case series substantiate further *in vivo* studies of H-FIRE as a clinical tool for the treatment of surface lesions in horses and potentially humans.

We have demonstrated that H-FIRE therapy is potentially an effective clinical procedure for the treatment and control of inoperable infiltrative superficial tumors in horses. The therapy can be delivered to horses that are awake, standing, and sedated, eliminating the need for general anesthesia and standard recumbent surgery that currently limit the use of conventional IRE and electrochemotherapy treatments. Therapy is delivered in a time-efficient manner (usually under 1 h including setup time) and post-treatment wound management is minimal and predictable. Repetitive treatments may be needed to control complex, large lesions but can be accomplished in an outpatient setting. Although multiple locations in large tumors may be treated during a single procedure, follow-up treatments seem to be necessary to maximize reduction in tumor size. Ultimately, we have shown that bursts of 2 μs pulses are effective for soft tissue ablation while mitigating muscle contractions without the need for chemical neuromuscular blockade. This novel therapy has the potential to expand the clinical role of IRE in veterinary patients and warrants further research.

## Ethics Statement

All study protocols were approved by the Virginia Tech Institutional Animal Care and Use Committee (protocol # 13-144-CVM) and informed consent for this experimental therapy was obtained from owners prior to enrollment/treatment.

## Author Contributions

All authors conceived of the study design, conducted experiments, analyzed data, and wrote and revised the manuscript.

### Conflict of Interest Statement

MD, RD, and JR have pending and issued patents in the area of electroporation. The remaining authors declare that the research was conducted in the absence of any commercial or financial relationships that could be construed as a potential conflict of interest.

## References

[1] Robertson J Diseases of the integumentary system. In: Rooney JR Robertson JL, editors. Equine Pathology. Iowa State University Press (1996). p. 287–307.

[2] Robertson JL Lymphosarcoma and other rare skin tumors. In: White NA Moore JN, editors. Current Techniques in Equine Surgery and Lameness. Philadelphia, PA: W. B. Saunders Co. (1997). p. 187–97.

[3] Cemazar M Tamzali Y Sersa G Tozon N Mir LM Miklavcic D. Electrochemotherapy in veterinary oncology. J Vet Intern Med. (2008) 22:826–31. 10.1111/j.1939-1676.2008.0117.x 18537879

[4] Souza C Villarino NF Farnsworth K Black ME. Enhanced cytotoxicity of bleomycin, cisplatin, and carboplatin on equine sarcoid cells following electroporation-mediated deliver *in vitro*. Vet Pharm Ther. (2016) 40:97–100. 10.1111/jvp.12331 27287308

[5] Tamzali Y Teissie J Golzio M Rols MP Electrochemotherapy of equine cutaneous tumors: a 57 case retrospective study 1999-2005. In: 11th Mediterranean Conference on Medical and Biomedical Engineering and Computing 2007. IFMBE Proceedings. Vol. 16 Berlin; Heidelberg: Springer (2007). p. 610–3.

[6] Tamzali Y Borde L Rols MP Golzio M Lyazrhi F Teissie J. Successful treatment of equine sarcoids with cisplatin electrochemotherapy: a retrospective study of 48 cases. Equine Vet J. (2012) 44:214–20. 10.1111/j.2042-3306.2011.00425.x 21793876

[7] Tozon N Kramaric P Kos Kadunc V Sersa G Cemazar M. Electrochemotherapy as a single treatment or adjuvant treatment to surgery of cutaneous sarcoid tumors in horses: a 31-case retrospective study. Vet Rec. (2016) 179:627. 10.1136/vr.103867 27758950

[8] Spugnini EP D'Alterio GL Eng ID Eng TM Dragonetti E Murace R Electrochemotherapy for the treatment of multiple melanomas in a horse. J Equine Vet Sci. (2011) 31:430–3. 10.1016/j.jevs.2011.01.009

[9] Miklavcic D Pucihar G Pavlovec M Ribaric S Mali M Macek-Lebar A The effect of high frequency electric pulses on muscle contractions and antitumor efficacy in vivo for a potential use in clinical electrochemotherapy. Bioelectrochemistry. (2005) 65:121–8. 10.1016/j.bioelechem.2004.07.004 15713562

[10] Davalos RV Mir LM Rubinsky B. Tissue ablation with irreversible electroporation. Ann Biomed Eng. (2005) 33:223–1. 10.1007/s10439-005-8981-8 15771276

[11] Yarmush ML Golberg A Sersa G Kotnik T Miklavcic D. Electroporation-based technologies for medicine: principles, applications, and challenges. Ann Rev Biomed Eng. (2014) 16:295–320. 10.1146/annurev-bioeng-071813-104622 24905876

[12] Martin RC II McFarland K Ellis S Velanovich V. Irreversible electroporation in locally advanced pancreatic cancer: potential improved overall survival. Ann Surg Oncol. (2013) 20:443–9. 10.1245/s10434-012-2736-1 23128941

[13] Arena CB Sano MB Rossmeisl JH Jr Caldwell JL Garcia PA Rylander MN. (2011) High-frequency irreversible electroporation (H-FIRE) for non-thermal ablation without muscle contraction. Biomed Eng online. (2011) 10:102. 10.1186/1475-925X-10-102 22104372 PMC3258292

[14] Latouche EL Arena CB Ivey JW Garcia PA Pancotto TE Pavlisko N. High-frequency irreversible electroporation for intracranial meningioma: a feasibility study in a spontaneous canine tumor model. Technol Cancer Res Treat. (2018) 17:1–10. 10.1177/1533033818785285 30071778 PMC6077896

[15] Jiang CL Davalos RV Bischof JC. A review of basic to clinical studies of irreversible electroporation therapy. IEEE Trans Biomed Eng. (2015) 62:4–20. 10.1109/TBME.2014.2367543 25389236

[16] Valentine BA. Equine melanocytic tumors: a retrospective study of 53 horses (1988 to 1991). J Vet Intern Med. (1995) 9:291–7. 10.1111/j.1939-1676.1995.tb01087.x 8531173

[17] Johnson PJ Dermatologic tumors of horses (excluding sarcoids). Vet Clin North Am Equine Pract. (1998) 14:625–58, viii. 10.1016/S0749-0739(17)30190-6 9891728

[18] Marti E Lazary S Antczak DF Gerber H. Report of the first international workshop on equine sarcoid. Equine Vet J. (1993) 25:397–407. 10.1111/j.2042-3306.1993.tb02981.x 8223371

[19] Knottenbelt DC Kelly DF The diagnosis and treatment of periorbital sarcoid in the horse: 445 cases from 1974-1999. Vet Ophthalmol. (2000) 3:169–191. 10.1046/j.1463-5224.2000.00119.x 11397301

[20] Cockerell GL MacCoy DM. Clinicopathological manifestations of selected neoplasms. Cornell Vet. (1978) 68:133–50. 204450

[21] Elbahi A Kipar A Ressel L. Histocytic-like atypicsl mast cell tumours in horses. J Comp Pathol. (2018) 162:14–17. 10.1016/j.jcpa.2018.05.003 30060838

[22] Kodre V Cemazar M Pecar J Sersa G Cor A Tozon N Electrochemotherapy compared to surgery for treatment of canine mast cell tumors. In Vivo. (2009) 23:55–62. 19368125

[23] Senior JM. Morbidity, mortality, and risk of general anesthesia in horses. Vet Clin North Am Equine Pract. (2013) 29:1–18. 10.1016/j.cveq.2012.11.007 23498043

[24] Golberg A Rubinsky B. Towards electroporation based treatment planning considering electric field induced muscle contractions. Technol Cancer Res Treat. (2012) 11:189–201. 10.7785/tcrt.2012.500249 22335414

